# Engineered Human Contractile Myofiber Sheets as a Platform for Studies of Skeletal Muscle Physiology

**DOI:** 10.1038/s41598-018-32163-1

**Published:** 2018-09-17

**Authors:** Hironobu Takahashi, Tatsuya Shimizu, Teruo Okano

**Affiliations:** Institute of Advanced Biomedical Engineering and Science, Tokyo Women’s Medical University, Tokyo, Japan

## Abstract

Skeletal muscle physiology and the mechanisms of muscle diseases can be effectively studied by an *in-vitro* tissue model produced by muscle tissue engineering. Engineered human cell-based tissues are required more than ever because of the advantages they bring as tissue models in research studies. This study reports on a production method of a human skeletal myofiber sheet that demonstrates biomimetic properties including the aligned structure of myofibers, basement membrane-like structure of the extracellular matrix, and unidirectional contractile ability. The contractile ability and drug responsibility shown in this study indicate that this engineered muscle tissue has potential as a human cell-based tissue model for clinically relevant *in-vitro* studies in muscle physiology and drug discovery. Moreover, this engineered tissue can be used to better understand the relationships between mechanical stress and myogenesis, including muscle growth and regeneration. In this study, periodic exercise induced by continuous electrical pulse stimulation enhanced the contractile ability of the engineered myofibers and the secretion of interleukin-6 (IL-6) and vascular endothelial growth factor (VEGF) from the exercising myofibers. Since the physiology of skeletal muscle is directly related to mechanical stress, these features point to application as a tissue model and platform for future biological studies of skeletal muscle including muscle metabolism, muscle atrophy and muscle regeneration.

## Introduction

Tissue engineering enables the production of native-like tissues that can potentially be transplanted for use in regenerative medicine and *in-vitro* tissue models for biological studies and drug discovery^1–5^. The recent advances in induced pluripotent stem (iPS) cell technology is opening up an entirely new era of possibilities for tissue engineering^6–8^. The current level of iPS cell technology can be applied to all cell types for the development of human cell-based tissue models, and even for cells that can only be obtained sparingly from the human body. Animal models have long been the main approach for drug discovery and prediction of pharmacokinetics but they have limitations for tissue modeling needed to understand the mechanisms in the human body^9,10^. In addition, *in-vitro* human tissue models will reduce the use of experimental animals, which has become an ethical cornerstone in the fields of pharmaceutical and cosmetic development. A number of tissue engineering studies have already demonstrated the production of *in-vitro* human tissue models for liver, lung, and cardiac tissues^3,5,11–14^. However, tissue modeling needs to be improved so that engineered tissues can be produced that are as native-like as possible. For example, some kinds of native tissues such as muscle, tendon, and bone have a specific microstructure, which is a key component of its ability to function appropriately. To engineer these tissue microstructures, therefore, the architecture of artificial tissue needs to closely mimic that of native tissues. Tissue engineering is an attractive approach to artificially recreate theses native tissue-like microstructures.

Muscle tissue engineering can now produce artificial muscle tissues that have the potential to help us better understand myogenesis including development, growth, and regeneration^15–17^. Since skeletal muscles also contribute to metabolic, neuromuscular, and dystrophic disorders, engineered muscle tissues will become a powerful tool to understand the mechanisms of these diseases and facilitate the discovery of new drugs for their treatment^18,19^. In native tissues, skeletal muscle has a highly oriented structure made of parallel bundles of muscle fibers and this architecture is known to be a key factor for producing the mechanical functions in native skeletal muscle. With this structure in mind, various studies have reported innovative strategies to produce structurally biomimetic muscle tissues^15,20–25^. We have also reported that aligned myotube constructs can be produced using a micropatterned substrate that allows regulation of cell orientation^26,27^. However, most studies produced rodent muscle tissues, while human cell-based tissues are required in the field of tissue modeling for an accurate understanding of the complex physiological phenomena in the human body. Functional human cell-based muscle tissue remains difficult to recreate in normal culture systems. In fact, although we succeeded in the production of human myotubes based on our own strategy, it was impossible to functionalize the muscle tissue simply by long-term culturing on a micropatterned substrate. Several previous studies overcame this issue by developing defined culture systems specifically for maturing myotubes^28,29^. For example, Bursac and co-workers reported a well-organized culture system that succeeded in the production of a functional muscle tissue with a biomimetic structure^30,31^. Although techniques such as these are crucial for producing a human tissue model of skeletal muscle, there have been very few significant approaches reported.

Here, we report on the basic biological studies of a functional human skeletal muscle tissue construct, a “myofiber sheet” using a micropatterned thermoresponsive culture substrate. Our group has developed a thermoresponsive cell culture substrate allowing harvest of a sheet-shaped cellular assembly, called a “cell sheet”, from the substrate^32–34^. The thermoresponsive polymer, poly(*N*-isopropylacrylamide) (PIPAAm), grafted on a culture substrate becomes more hydrophilic by simply lowering the culture temperature to 20 °C. This change induces the detachment of intact confluent cells as a single continuous cell sheet from the culture surface. Harvesting without the use of any enzymatic treatment means that cell sheets can be transferred while maintaining the sheet-shaped assembly, including their cell-cell junctions. To date, this system has been effectively used for a number of significant achievements in regenerative medicine^35–37^. In this study, our cell manipulation technique was used to develop a culture system for the functionalization of human muscle tissues. The muscle tissues produced using this culture method exhibits potential for use as an effective tissue model based on the results of its contractile ability and drug responsibility.

## Results

### Fabrication of Human Muscle Tissue Construct “Myofiber Sheet”

**Figure 1:**
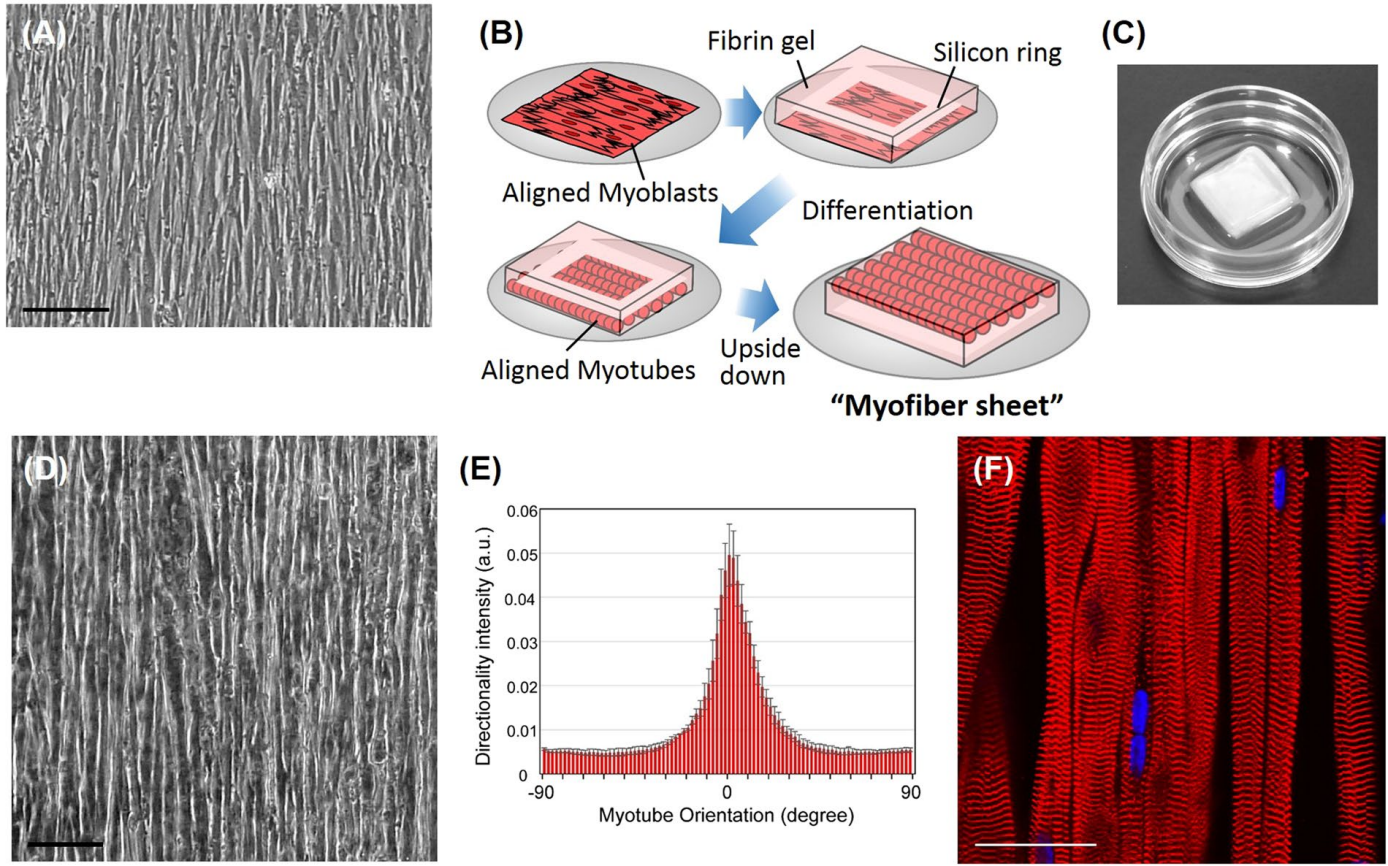
(**A**) Microscopic image of aligned myoblasts on the micropatterned surface. (**B**) Schematic illustration of the production procedure of a myofiber sheet. Aligned myoblasts are transferred from the micropatterned surface to the fibrin-based gel. A silicon ring was incorporated in the gel to prevent shrinkage of the gel sheet. After 1 week of incubation in differentiation medium, the myofiber sheet detached with the gel from the surface. (**C**) Photograph of a myofiber sheet incorporated in a square-shaped fibrin gel (size: 15 × 15 mm; depth: approx. 4.2 mm). (**D**) Phase contrast microscopic image of a myofiber sheet in fibrin gel at 3 weeks after induction of differentiation. (**E**) Distribution of myotube orientations on the fibrin-based gel at 3 weeks after induction of differentiation. (n = 3) (**F**) Confocal microscopic image of a myofiber sheet showing sarcomere structures in the gel. Sarcomeric α-actinin (red) and nuclei (blue) were stained fluorescently. Scale bar: (**A,D**) 100 μm, (**F**) 50 μm.

To engineer a myofiber sheet, human skeletal muscle myoblasts were initially seeded onto the micropatterned thermoresponsive surface^26,27,38^. As shown in Fig. 1A, myoblasts were aligned with the direction of the patterns, and reached confluence while maintaining their orientation. After reaching a confluence, human dermal fibroblasts were then seeded onto the myoblast sheet for co-culturing with the myoblasts. Both cell types were similarly aligned and formed a single continuous cell sheet. To confirm that the patterned surface allows the cells to be released from the surface by decreasing culture temperature, the aligned cells were incubated at 20 °C for 30 min at Day 5, in the absence of a fibrin gel. In this case, the aligned cells detached freely from the surface as a single continuous cell sheet. However, this cell sheet still needed to be cultured in an appropriate environment for maturation. To produce a myofiber sheet, fibrinogen was mixed with thrombin and Matrigel, and then poured onto the co-cultured cell sheet on the day following seeding of fibroblasts onto the myoblast sheet. After the formation of the Matrigel-containing fibrin-based gel (Fib/Mtr gel) on the cell sheet, they were cultured in a differentiation medium. After 1 week of culture, the temperature was lowered to 20 °C, and the aligned cells were harvested with the gel from the culture surface (Fig. 1B,C). Importantly, the aligned orientation of myoblasts was maintained through the transfer of the cells from the patterned surface to the Fib/Mtr gel, and differentiation into myotubes occurred after a further 2 weeks (Fig. 1D,E). When myotubes were cultured on the surface without the Fib/Mtr gel, they detached spontaneously from the surface during the 3 week-culture. In contrast, this culture method allows human myotubes to be cultured for a sufficiently long period required for maturation into contractile myofibers. As a result, sarcomere structures were observed in the aligned myofibers (Fig. 1F). During the maturation stage, the developing myofibers generated a force that shrank the Fib/Mtr gel along the direction of the cell alignment. Therefore, in all samples, a silicon ring was incorporated in the gel that successfully prevented any shrinkage and maintained the shape of the gel sheet (Fig. 1B,C). In addition, when the myofibers were observed using confocal microscopy at high magnification, their vertical positions were slightly different to each other in the gel. This confirmed that they had slightly migrated vertically during differentiation, and that the myofibers were finally incorporated into the Fib/Mtr gel as shown in the illustration of Fig. 1B.

### Contractile Ability of Aligned Myofiber Sheet

**Figure 2:**
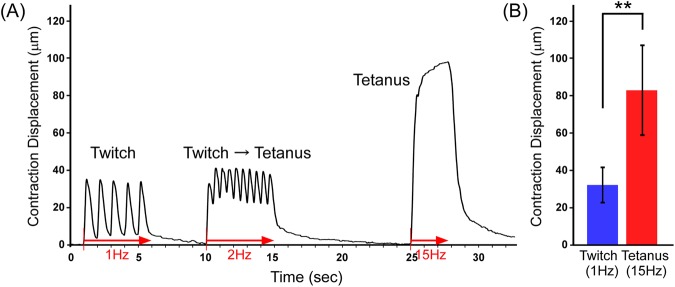
(**A**) Representative displacement profile of a myofiber sheet stimulated electrically at various frequencies (1, 2, and 15 Hz). This profile shows that the myofiber sheets showed a twitch contraction at 1 Hz and the contraction was partially fused at 2 Hz. At 15 Hz, myofibers shortened more dynamically (approx. 100 μm) due to the tetanic contraction. The arrows indicate the time period of EPS application at 1, 2, and 15 Hz. (**B**) Displacement of twitch and tetanic contractions at frequencies of 1 Hz and 15 Hz, respectively. (n = 3) (**P < 0.01).

In order to evaluate the functionality of the myofiber sheets, they were stimulated electrically, and the contractile behavior was observed microscopically. As shown in Supplementary Video 1, muscle contraction coincided with the frequency of electrical pulse stimulation (EPS). Importantly, the myofiber sheet contracted unidirectionally due to the myoblast orientation created using the micropatterned culture substrate. This contractile behavior is required for an engineered muscle tissue to mimic the functionality of native skeletal muscles. In addition, this tissue construct showed that muscle contractions were dependent on the EPS conditions. When the myofiber sheet was simulated at a frequency of 0.5 or 1.0 Hz, it produced a twitch contraction (Fig. 2, Supplementary Video 1). On the other hand, as the frequency increased from 1.0 to 2.0 Hz, the contraction was partially fused, and the contraction baseline moved up. This fusion of twitch contractions is mainly caused by the physiological temporal summation phenomena of the skeletal muscles. Moreover, the stimulation at a frequency of 15 Hz induced a tetanic contraction, and the myofibers shortened more dynamically. As shown in Fig. 2B, the displacement of the tetanic contractions was significantly higher than that of the twitch contractions. These contractile behaviors, which depended on the EPS frequency, indicated that the myofiber sheet was physiologically functional.

Furthermore, the contractile ability was maintained even after 2 months (n = 3, the representative movie shown in Supplementary Video 2). That is, the myofiber sheet can function stably over a long period of time. On the other hand, there was no increase in the contractile ability during the culture period over several months. This might suggest that there is a limit in long-term culture to enhance the contractile ability.

### Anisotropic ECM formation surrounding aligned myofibers

**Figure 3:**
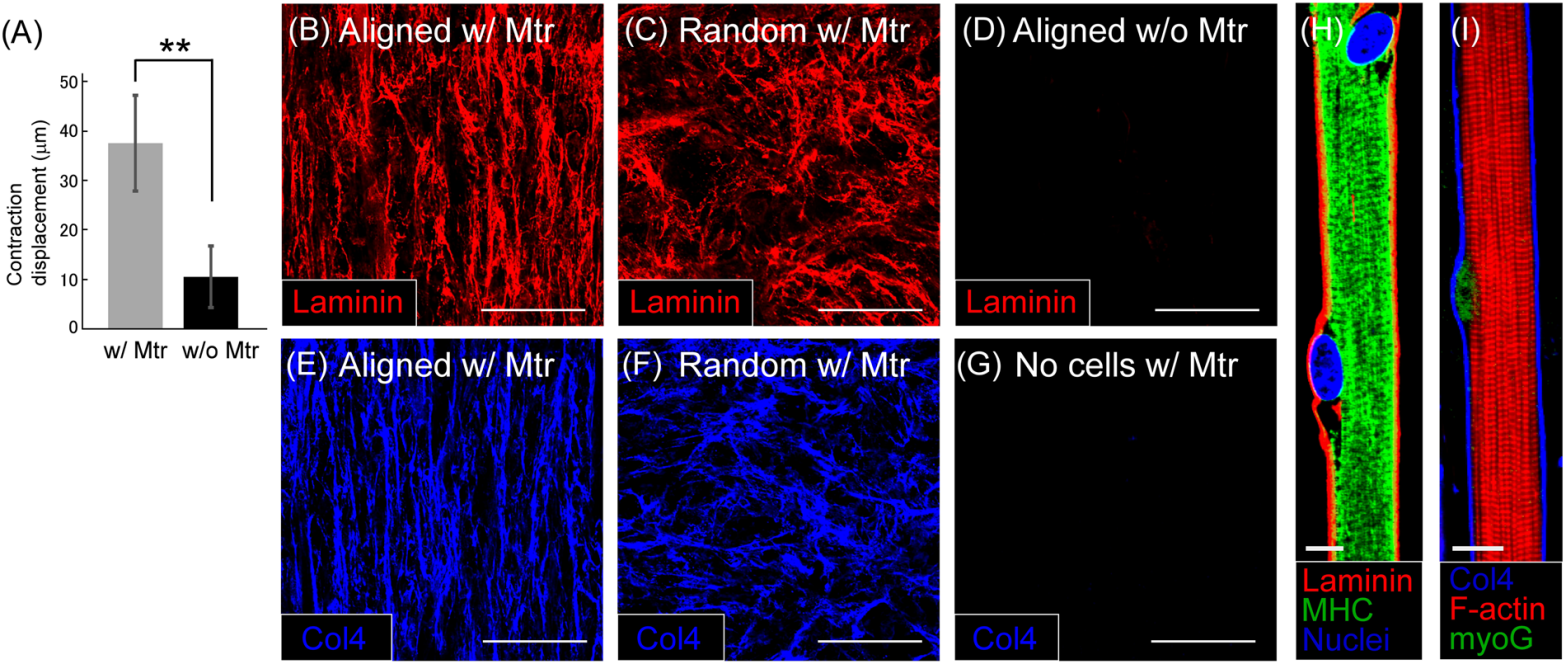
(**A**) Comparison of contraction displacement between myofiber sheets cultured in the fibrin-based gels with (w/ Mtr) and without Matrigel (w/o Mtr). (n = 5) (**P < 0.01) (**B,C**) Laminin structure shown in the gel containing Matrigel (w/ Mtr). Myofibers are aligned **(B)** or randomly oriented in the gel **(C)**. (**D**) No laminin formation observed in the gel without Matrigel (w/o Mtr). The aligned cells produced no laminin structure in the gel. (**E,F**) Formation of anisotropic and isotropic structures of type IV collagen in the Matrigel-containing gels with **(E)** aligned and **(F)** randomly oriented myofibers. (**G**) No formation of type IV collagen in the gel containing Matrigel, but without cells. (**H**) Laminin surrounding a myofiber in the myofiber sheet. (red: laminin, green: myosin heavy chain (MHC), blue: nuclei) (**I**) Type IV collagen surrounding a myofiber (blue: Type IV collagen, red: F-actin, green: myogenin (myoG)) Scale bars: 100 μm (**B–G**), 10 μm (**H,I**).

Matrigel has been widely used in a number of tissue engineering studies and some research groups have demonstrated that this ECM mixture was useful in tissue engineering^14,30,31,39,40^. In this study, it was expected that Matrigel could improve maturation of myotubes when mixed in the fibrin gel. To confirm its affect, aligned myoblasts were transferred to fibrin gel with (Fib/Mtr) or without Matrigel (Fib/-Mtr). The absence of Matrigel resulted in fewer myotubes to remain on the Fib/-Mtr gel at 3 weeks compared to that on Fib/Mtr gel, and the contractile ability was significantly improved from that on Fib/Mtr gel. In fact, the contracting displacement of myofiber sheets on the Fib/-Mtr gel decreased to 28.0% of the displacement on the Fib/Mtr gel (Fig. 3A). This clearly indicates that the addition of Matrigel effectively promoted the functionality of the myofibers when mixed in the gel. In fact, a previous study reported by the Bursac group also used this matrix mixture to produce contractile muscle tissue constructs^31^.

More interestingly, fluorescence images showed that laminin formed an anisotropic structure in the Fib/Mtr gel (Fig. 3B). The anisotropy was in the same direction as the myofibers in the gel. Using a non-patterned surface, a randomly oriented myofiber sheet can also be produced. In this case, the ECM protein formed a random structure due to the random orientation of the cells (Fig. 3C). These results indicate that the anisotropy in the laminin structure is caused by the orientation of the myofibers. On the other hand, in the gel without Matrigel (Fib/-Mtr gel), no laminin structures formed, as seen in the Fib/Mtr gel (Fig. 3D). Therefore, the presence of Matrigel was necessary for the laminin formation. In addition, type IV collagen also formed a structure similar to laminin in the gel (Fig. 3E,F). Both laminin and type IV collagen are known to be the main components of Matrigel. However, these ECM proteins forming the anisotropic structures did not originate from the Matrigel itself. When no cells were cultured in Fib/Mtr gel, neither of the ECM proteins formed anisotropic structures in the gel (Fig. 3G). Therefore, it is reasonable to conclude that mature myotubes created this ECM formation by themselves. Otherwise, they might have organized the ECM proteins present in Matrigel to form an anisotropic structure during their maturation. Moreover, the laminin and type IV collagen were observed surrounding the myofibers (Fig. 3H,I). As previously reported, this basement membrane-like structure is also found in native skeletal muscles^30,31^. This biological aspect probably influences the maturation of the myofiber sheet. Taken together, the myofibers produced laminin and type IV collagen and the ECM proteins formed the unique structure surrounding the myofibers in the gel. However, the Matrigel was also necessary for the formation of the ECM structure in the gel. Although the anisotropic ECMs were not made from Matrigel, Matrigel did trigger the cells to produce the basement membrane-like ECM proteins, resulting in the promotion of maturation^41^.

### Drug responsibility of human myofiber sheet construct

**Figure 4:**
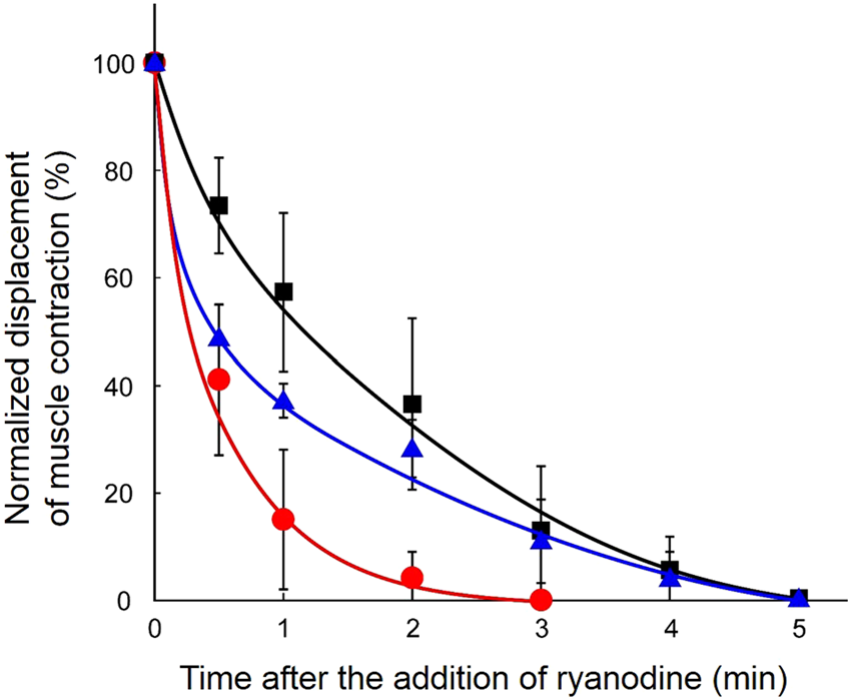
Profiles of muscle contraction suppressed by the addition of ryanodine (black square: 50, blue triangle: 100, and red circle: 200 μM). The displacement was normalized to that just after the addition of ryanodine (time = 0 min) as 100% and indicates the rate of decrease (n = 4). EPS (frequency: 1 Hz) was continuously applied in these experiments.

To demonstrate the potential for drug development, responses of the myofiber sheet to a pharmaceutical agent were investigated. In this study, ryanodine, a well-known modulator of ryanodine receptor (RyR)-mediated Ca^2+^ release, was used as a representative agent affecting muscle contraction^42^. This agent was expected to influence muscle contraction through the specific interaction of ryanodine with RyR in the myofibers, and was actually significantly affected by the addition of ryanodine (Fig. 4). With 50 μM of ryanodine in the medium, the contracting displacement was decreased to approx. 60% after 1 min. Although the myofiber sheet was continuously stimulated (10 V voltage, 1 Hz frequency, 3 ms duration), contraction and relaxation of the myofibers completely stopped within 5 min (Supplementary Video 3). In addition, higher doses of ryanodine more effectively inhibited the EPS-induced contraction and relaxation. For example, by increasing the ryanodine concentration to 200 μM, contracting displacement decreased to less than 20% after 1 min. These results suggest that ryanodine physiologically affected muscle contraction through its specific interaction with RyR in the myofibers. In the physiology of native muscle tissues, ryanodine reacts to RyR present on the membrane of sarcoplasmic reticulum and then induces the release of Ca^2+^ from the sarcoplasmic reticulum. The results shown in Fig. 4 indicate that the myofiber sheet construct produced in this study has a physiological mechanism similar to that found in native skeletal muscles. That is to say, the myofiber sheets were functionally similar to native muscle tissues and this allowed us to validate their potential use in future predictive studies of muscle physiology.

### EPS-induced exercise effect on myofiber maturation

**Figure 5:**
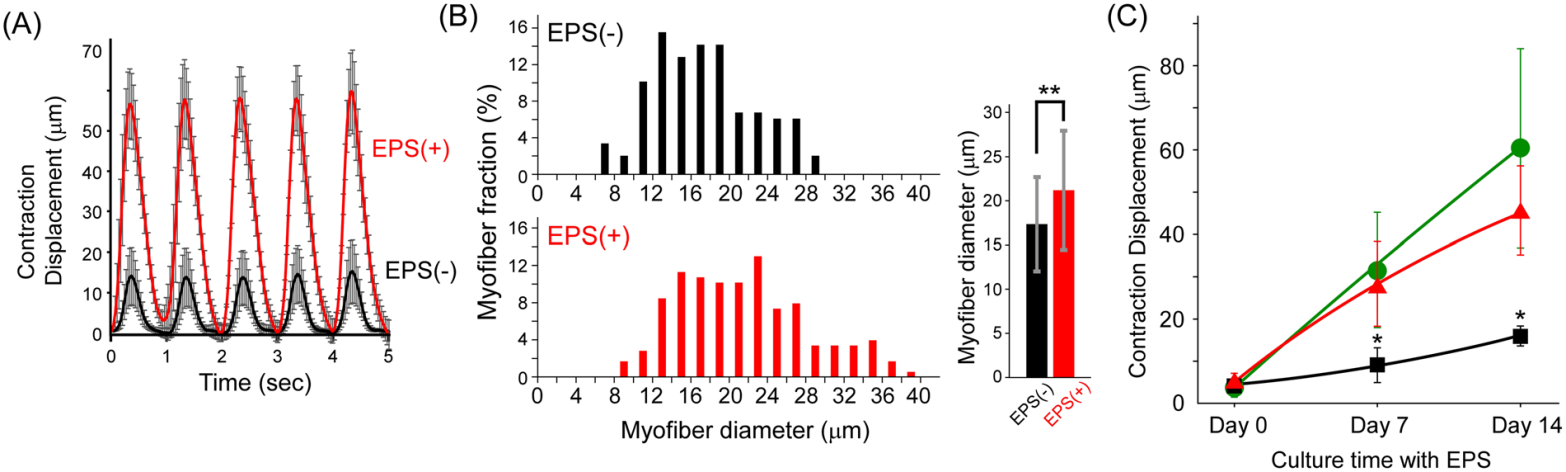
Effect of EPS-induced continuous contraction on maturation of myofiber sheets. (**A**) Contraction displacement of myofiber sheets cultured with no stimulation for 10 days (EPS(−)) and with no stimulation for 7 days and then continuous stimulation for 3 days (EPS(+)). Continuous EPS (voltage: 10 V, frequency: 1 Hz, duration: 3 ms) was applied for 1 h with 3 h rest. This procedure started at day 7 after induction of differentiation and was repeated for 3 days. To compare their contraction abilities, EPS was applied at 1 Hz frequency for 5 seconds. (n = 3) (**B**) Distribution and average value of myofiber diameters with and without continuous EPS for 2 weeks. The data were summarized from three myofiber sheets for each group (total number of myofibers: more than 150). (**P < 0.01) (**C**) Effect of continuous EPS on contractile ability of myofiber sheets for 2 weeks. Two different continuous EPS (triangle: 3 ms, circle: 10 ms) were applied for 1 h with 3 h rest. This procedure started at day 7 after induction of differentiation (Day 0 for EPS exercise) and was repeated for 2 weeks (Day 14). As the control, myofiber sheets were cultured without continuous EPS (square). The displacements of the shortening myofibers were monitored at Day 0, 7, and 14. (n = 5) (*P < 0.05 vs other samples at the same time point).

It is well known that muscle maturation is related with various mechanical stresses in the body. To investigate the effect of contraction-induced stress on myofiber sheets, EPS was continuously applied to the myofiber sheets for 2 weeks. In this study, EPS (10 V voltage, 1 Hz frequency, 3 ms duration) was applied continuously for 1 h and then rested for 3 h. This procedure started at 1 week after induction of differentiation and repeated for 2 weeks. The effect of this EPS-induced exercise was observed several days after application. When the myofiber sheets were stimulated to determine their contractile ability, the muscle contractions were enhanced after only 3 days of exercise (Fig. 5A, Supplementary Video 4). Furthermore, after 2 weeks of EPS-induced exercise, the myofiber sheets were more matured, compared with the same myofiber sheets cultured for 2 weeks without the EPS-induced exercise. The EPS-induced exercise triggered an increase in the average diameter of the myofibers (EPS(+): 21.2+/−6.7 μm, EPS(−): 17.3+/−5.3 μm). In addition, the distribution of myofiber diameter indicated that the exercise for 2 weeks promoted myofiber hypertrophy in partial myofibers (Fig. 5B). Whereas even when cultured with no electrical stimulation, the myofibers did gradually mature by 3 weeks after induction of differentiation; however, the contractile ability of the exercised myofiber sheets was much higher (Fig. 5C). In addition, to investigate the difference that exercise had on maturation of myofiber sheets, a continuous EPS with a duration of 10 ms was also applied. Since the longer duration time caused more dynamic contraction of myofibers compared with a shorter duration, the exercise effect at a duration of 10 ms was expected to have a large influence on the functionality of myofiber sheets, compared with a duration of 3 ms. However, as shown in Fig. 5C, the exercise effects of 3 ms and 10 ms produced the same level of muscle contraction. Taken together, the exercise induced by EPS at a duration of 3 ms was sufficiently effective to enhance the functionality of the myofiber sheets in this study. The relationship between muscle exercise and functionality (maturation) is an important topic in muscle physiology and the study of muscle regeneration. These results suggest that this engineered tissue construct can be used for basic biological studies in this field.

### Effects of EPS-induced exercise on cytokine secretion by contracting myofibers

**Figure 6:**
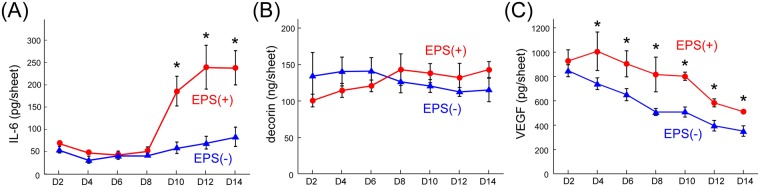
Profiles of cytokine secretion from myofiber sheets with (EPS(+)) and without continuous electrical stimulation (EPS(−)) ((**A**) IL-6, (**B**) decorin, (**C**) VEGF). At Day 7 after induction of differentiation, EPS application (voltage: 10 V, frequency: 1 Hz, duration: 3 ms) was started (Day 0), and culture media were collected every other day for 2 weeks (from Day 2 to Day 14). Representative profiles are shown (n = 3). The significant difference between EPS(+) and EPS(−) groups was considered at each day (*P < 0.05 at the same time point).

In general, it is known that skeletal muscles in the body secrete various kinds of cytokines, so-called myokines, during adequate contraction. In this study, while EPS was applied repeatedly for 2 weeks, conditioned media were collected to determine the number of cytokines released by the contracting myofibers. As shown in Fig. 6A, the secretion of interleukin-6 (IL-6) was markedly increased by the sequential muscle contraction. Fig. 6A shows a representative profile, but the time point when IL-6 secretion increased shifted slightly in each experiment. For example, in other cases, the secretion started increasing at Day 6. That is, although the time point of increasing IL-6 secretion cannot be identified, the secretion of IL-6 was obviously promoted by the exercise of the myofiber sheets. In addition, since a previous study reported that cytokines are regulated by skeletal muscle contraction, the expression of decorin was also investigated through the same procedure^43^. However, the secretion was not enhanced by the continuous muscle contraction (Fig. 6B). Although the reason why the secretion was not enhanced by the muscle contraction was not elucidated, these results do indicate that this myofiber sheet can be used for basic biological studies in understanding autocrine and paracrine effects of contraction-induced cytokines on muscle maturation, metabolism, and regeneration.

The EPS-induced exercise in this study possibly affected the secretion of cytokines produced by fibroblasts, since the fibroblasts in the myofiber sheet were not stimulated electrically but physically during the continuous contraction. In fact, vascular endothelial growth factor (VEGF) released from the myofiber sheet was also increased by the EPS-induced muscle contractions (Fig. 6C). Although fibroblasts normally secret VEGF regardless of any physical stimulation, the muscle contractions significantly enhanced the secretion. Importantly, this also suggests that the physical stimulation to the co-cultured fibroblasts may have increased the production of some other cytokines. In addition to the physical stimulation, it is possible that some myokines released by contracting myofibers may have influenced the co-cultured fibroblasts, and then the paracrine effects induced a change in some cytokine secretions from the fibroblasts.

## Discussion

A number of studies have reported that formation of myotubes can be achieved simply by culturing myoblasts in differentiation medium (e. g., 2% horse serum containing medium) onto a normal culture dish (e.g., tissue culture polystyrene (TCPS) dish). However, this normal 2D culture system does not allow human myotubes to mature sufficiently to develop a significant contractile ability. For example, in our study, although long-term culture is required for sufficient maturation of a human muscle tissue, most of the human myotubes produced on the patterned surface generated a force during the maturation stage and then spontaneously detached from the surface. Therefore, most studies in muscle tissue engineering have only been able to report the production of mouse models. Although some advanced techniques have been previously developed to provide an improved environment for myogenic cells, most of these studies reported to produce aligned mouse myotube constructs^44,45^. Moreover, these aligned myotubes still need to be functionalized for development of *in-vitro* tissue models, but it remains very difficult to functionalize human myotubes. Since *in-vitro* tissue models can contribute to advances in drug discovery studies, a wide range of muscle tissue models have been adapted for drug testing studies^20,30,46^. However, to better understand the effects of drug candidates in the human body, human cell-based tissue models are required. In this study, a unique culture system has been developed to produce structural regulation and functional maturation of human myofibers. The aligned structure of these engineered myofibers was regulated using a micropatterned substrate, and then transferred onto a fibrin-based gel for further maturation. They were able to attach to the gel and further cultured, while maintaining their cell orientation for the long term (greater than one month). Ultimately, aligned functional myofibers were successfully produced in the gel using this tissue engineering method.

The engineered myofibers also showed a native tissue-like ECM structure in the gel. When Matrigel is added to the gel it plays an important role in the formation of the structure of laminin and type IV collagen. In native muscle tissue, myofibers are surrounded by a basement membrane which also contains laminin and type IV collagen and it is well known that this basement membrane is necessary for mature myofibers in the body. Since the myofibers in this study were similarly surrounded by laminin and type IV collagen, this ECM structure is an indicator of myofiber maturation. A previous study also reported that Matrigel was used to accelerate maturation of myofibers^41^. The Bursac group reported that Matrigel-containing fibrin gel was effectively used to produce mature myofibers based on their tissue engineering method^30,31^. Considering this biological characteristic, it could become a useful tissue model for studies of muscle diseases, such as muscle dystrophy.

The fibrin-based gel also provides myofibers with an elastic environment suitable for muscle contraction. The elasticity supports flexible displacement during the contraction of myofibers. In addition, although the maximum contractile force that the tissue can exert has not yet been quantified, the physiological characteristics of the myofiber sheet are such that this tissue construct can be used as a functional muscle tissue model. Furthermore, the EPS-induced muscle contraction was prevented chemically by the addition of ryanodine, a compound relating to calcium ion channels (Fig. 4). The drug responsibility was also preliminarily tested using nifedipine, a calcium ion channel blocker. These indicate that the myofiber sheet can be used for preclinical drug testing. Using disease-specific iPS cell-derived myoblasts, and this approach, patient-specific customized tissue models are also possibly in the future. It is believed that creating a disease-specific model will be very useful for understanding the specific disease mechanisms and drug discovery for its treatment^47–49^. In order to effectively apply an engineered tissue construct as an *in-vitro* tissue model, a new analysis system will be important. In this study, contraction displacement was analyzed using a motion analysis tool, similar to previous studies^24,25^. This measurement was useful to display the physiological responses of the myofiber sheet construct. However, a standard measurement system for contractile force that can be adjusted for the tissue model produced in this study needs to be developed to quantitatively examine tissue functionality in future studies.

Skeletal muscles are subjected to mechanical stress in the body and thus myogenesis is regulated by several kinds of mechanical stresses. In some basic studies, mechanical stimulation such as cyclic stretching effectively enhanced maturation of engineered muscles^23,50,51^. In contrast to mechanical stimulation, the EPS-induced contraction is a physiological procedure which mimics more closely muscle exercise. In fact, some studies reported that electrical stimulation influenced muscle growth, differentiation and maturation^23,52,53^. For example, a previous study reported by Ito *et al*. demonstrated that various EPS conditions including voltage, pulse width and frequency are related to the exercise effect in maturation of mouse myotubes^52^. Since the electrical stimulations in this study significantly enhanced the contractile ability of the human myofiber sheet (Fig. 5), the EPS-induced exercise procedure was effective in producing more mature and highly functional human myofibers. Appropriate mechanical stress is also necessary to activate the mechanism of muscle regeneration after muscle injuries and maintaining the functionality of skeletal muscle in the body. From this point of view, this tissue construct could also be applied as a tissue model for studying the relationship between exercise (mechanical stress) and muscle growth and regeneration.

Skeletal muscle is recognized as a major endocrine organ releasing myokines that are essential for the communication between muscle and other tissues such as adipose tissue, liver, and pancreas^54,55^. Many proteins produced by skeletal muscle are correlated with muscle contraction, and some specific myokines released from contracting muscle might mediate the health-promoting effects of physical activity^54^. For example, IL-6 and IL-15 are involved in muscle hypertrophy and myogenesis, and are known to have systemic effects on the liver, adipose tissue and the immune system. It has been reported that myostatin, follistatin and IL-7 are involved in the regulation of muscle mass in response to exercise. For studies of muscle physiology, therefore, it is important to understand the roles of these cytokines in the body. IL-6 is well known to be produced by contracting myogenic cells in response to exercise^56,57^, and it was reported that locally released IL-6 promotes an increase in muscle mass^58,59^. This study demonstrated that the myofiber sheet could be used to investigate the relationship between exercise-induced IL-6 secretion and muscle maturation. Because of the highly complex interrelated stimuli evoked by muscle contractions, physiological responses of contracting muscle have mainly been assessed using exercising animal models and human subjects. Although in this study only the increase in IL-6 secretion was confirmed, this human muscle tissue model will be an effective new tool to investigate relationships between the effects of exercise-induced cytokines and their related chronic diseases. Exercise with adequate physical activity is well known to be important for health by preventing the development of many chronic diseases such as obesity, type 2 diabetes, and sarcopenia^60–62^. These results indicate that this tissue construct will become a useful tissue model for broad applications in the study of muscle physiology.

Since fibroblasts comprise more than 50% of the extramuscular cell populations within skeletal muscle, they have an important role in skeletal muscle physiology. In fact, some previous studies reported that they promoted myotube differentiation in their *in-vitro* studies^63–65^. Here the effect of NHDFs co-cultured in the myofiber sheet was investigated. In this study, the co-cultured fibroblasts exhibited a mechanically-induced biological response (increased VEGF secretion with muscle contraction). Interestingly, several recent reports have shown that VEGF plays several important roles in skeletal muscle regeneration^66–69^. The regeneration after muscle injury is related with various physiological changes within fibroblasts, inflammatory cells and macrophages. In addition, some human training studies have demonstrated that acute exercise greatly elevated the VEGF mRNA level^70,71^. Therefore, VEGF might be a key factor not only in vascularization, but also in muscle regeneration. The increase in VEGF secretion may improve skeletal muscle repair by modulating angiogenesis, muscle regeneration and fibrosis through paracrine effects. Considering all these findings, the increase of VEGF from muscle contraction found in this study might provide a clue to better understand the important roles of fibroblasts in muscle regeneration. This also suggested that the myofiber sheet developed in this study can be useful as a tissue model for the studies focusing on cooperative physiology involving various kinds of cells in muscle regeneration.

## Summary

The culture system developed in this study enables the production of functional human muscle tissue constructs. The contractile ability and drug responsibility of this myofiber sheet demonstrate that this engineered muscle tissue can be used as a human cell-based tissue model for clinically relevant *in-vitro* studies of muscle physiology and drug development. In addition, this tissue model could possibly help us to better understand the mechanisms of muscle growth and regeneration in the human body. In this study, the EPS-induced exercise effectively promoted the maturation of myofibers and secretion of some kinds of cytokines were increased by the contracting muscle. Since functionality of skeletal muscle is related to mechanical stress including adequate contraction, this tissue model is expected to become a powerful tool for studies in the field of mechanobiology. Furthermore, this tissue modeling technique could be applied to engineer disease-specific muscle tissues using iPS cells from individual patients with the same muscle disease. A patient-specific tissue model should allow us to better understand the patient’s own condition and provide customized therapies.

## Methods

### Preparation of micropatterned thermoresponsive surface

The original procedure for creating polymer patterning on a thermoresponsive surface was reported previously^38,72^. In this study, to simplify the patterning procedure, a photo-induced polymerization method was adapted. To produce micropatterns from a hydrophilic polymer on a thermoresponsive surface, acrylamide (AAm) was polymerized using a photo-initiator and visible light onto a culture surface. A commercially available thermoresponsive culture dish, UpCell® dish (CellSeed, Inc., Tokyo, Japan), was used in this study. AAm aqueous solution (50 w/w%) (Wako, Tokyo, Japan) containing a photo-initiator water-soluble camphorquinone (7,7 dimethyl-2,3-dioxobicyclo[2.2.1] heptane-1-carboxylic acid) (1 w/w%) was poured into the substrate and visible light was irradiated for 7 min onto the substrate through an appropriate photomask. Sequentially, a hydrophilic polymer PAAm was grafted spatio-selectively as stripe-shaped micropatterns (the width of non-irradiation and irradiation regions: 50 μm) on a PIPAAm-grafted substrate.

### Production of a myofiber sheet incorporated in fibrin-based gel

Human skeletal muscle myoblasts at passage 1–3 (Lonza, Walkersville, MD, USA) were seeded at a density of 5 × 10^4^ cells/cm^2^ onto the patterned thermoresponsive substrate (the seeding area: 15 × 15 mm^2^) and then cultured in muscle cell growth medium (SkGM-2, Lonza) until reaching a 100% confluence. Next, normal human dermal fibroblasts (NHDFs) (Lonza) were further seeded onto the myoblast sheet at a density of 5 × 10^4^ cells/cm^2^. After the NHDFs adhered and spread on the myoblast sheet, a mixture of fibrinogen (from bovine plasma, 10 mg/mL, 2 mL) (Tokyo Chemical Industry, Tokyo, Japan), thrombin (from bovine plasma, 20 U/mL, 500 μL) (Sigma-Aldrich, St. Louis, MO, USA), Matrigel (500 μL) (BD Biosciences, San Jose, CA, USA) and CaCl_2_ solution (8 mM, 1 mL) was poured onto the cells (900 μL per a dish). Before the formation of the Matrigel-containing fibrin gel (Fib/Mtr gel), a squared-shaped silicon ring was inserted into the mixture to prevent shrinkage of the gel during the maturation of the myogenic cells. The culture dish was incubated for 30 min at 37 °C to allow the gel to form, then a differentiation medium with anti-fibrinolytic agents (20 μg/mL aprotinin or 2 mg/mL 6-aminocapronic acid (Sigma-Aldrich)) was added to the culture dish. In this study, Neurobasal medium with B27 supplement (Thermo Fisher Scientific, Waltham, MA, USA) was used to induce differentiation into myotubes^73,74^. After 1 week of culture in the differentiation medium, the aligned cells were incubated at 20 °C for 30 min to release them from the surface. The cell sheet attaching gel was placed upside down on the culture dish, and further cultured on the Fib/Mtr gel in the differentiation medium. To assess myotube orientation, a directionality histogram was constructed using the “directionality” function in the ImageJ software. The value of 0° denotes parallel alignment from the axis of the stripe patterns and 90° represents perpendicular alignment. Images with completely isotropic content will produce a flat histogram, whereas images in which there is a preferred orientation will produce a histogram with a clear peak at the respective orientation.

### Immunofluorescence staining

Cells encapsulated in the Fib/Mtr gel were fixed with 2% paraformaldehyde in PBS overnight at 4 °C. Following fixation, samples were washed with phosphate buffered saline (PBS) and then incubated in blocking solution (5% bovine serum albumin with 0.2% Triton-X 100) (Sigma-Aldrich) for 12 h^30,31^. The myofiber sheet with Fib/Mtr gel was treated with primary antibodies (sarcomeric α-actinin (1:500) (Abcam, Cambridge, MA, USA), laminin (1:500) (Abcam), type IV collagen (1:500) (Abcam), myosin heavy chain (1:200) (R&D Systems, Minneapolis, MN, USA), myogenin (1:500) (Thermo Fisher Scientific)) or AlexaFluor 568-conjugated phalloidin at 4 °C overnight. After washing with PBS, the tissue constructs were treated with fluorescently labeled secondary antibodies (1:800) (Thermo Fisher Scientific) for 2 h at 37 °C. For nuclei staining, tissue constructs were incubated with Hoechst33258 (Dojindo Laboratories, Kumamoto, Japan) for 5 min at RT. Images were acquired using a Zeiss 510 inverted confocal microscope and analyzed using LSM Image Software.

### Electrical pulse stimulation for muscle tissue construct

At seven days after the induction of differentiation, the aligned cells cultured with Fib/Mtr gel were harvested from the surface by lowering culture temperature (20 °C, 30 min) and then placed in 6-well plates for application of electrical pulse stimulation (EPS). Two carbon electrodes (C-Dish; IonOptix, Milton, MA, USA) were immersed into the medium, then the EPS was applied using an electrical pulse generator (IonOptix). To observe the contractile activity, the myofiber sheet was electrically stimulated (voltage: 10 V, frequency: 1, 2 or 15 Hz, duration time: 10 ms). To culture myofiber sheets with sequential stimulation, EPS (voltage: 10 V, frequency: 1 Hz, duration time: 3 or 10 ms) was applied for 1 h followed by incubation without stimulation for 3 h. This procedure started at 1 week after induction of differentiation and was carried out repeatedly for 2 weeks (from week 1 to week 3). The culture media were harvested for studies of cytokine production and replaced with fresh medium every other day.

### Microscopic contraction measurement

Myofiber sheets were observed microscopically and the EPS-induced muscle contraction was monitored sequentially using a CCD camera (Basler Ace, Basler AG, Germany; 30 frames/s) by phase contrast microscopy. Cell shortening distances were calculated using a motion analysis tool ViewPoint (Glenallan Technology Inc., Clinton, NY, USA)^24,25^. Even if the myofiber sheets were produced under the same culture conditions (on Fib/Mtr gel, in neurobasal medium, for 3 weeks), cell conditions (donor, passage number, seeding density etc.) also affected the functionality of tissue constructs^75^. In general, myoblasts freshly harvested from donors produce more highly functional myofibers, compared to myoblasts cultured *in vitro* for several passages. Therefore, in each experiment, the contractile ability was evaluated relatively, in comparison with the control samples prepared at the same time.

### Pharmacological treatments

In the pharmacological experiments, ryanodine (Sigma-Aldrich) was added to the medium at final concentrations of 50, 100 or 200 μM. Culture medium was replaced with the prepared medium and EPS (3 ms duration, 1 Hz frequency) was applied just after the media change. The muscle contraction was recorded until the twitch contraction was completely suppressed. The displacement of the contractions was calculated using ViewPoint.

### Cytokine production by contracting myofiber sheets

Culture media were collected at the indicated time points and the samples were analyzed by enzyme-linked immunosorbent assay (ELISA). ELISA (for interleukin-6 (IL-6), vascular endothelial growth factor (VEGF): R&D, for decorin: Abcam) was performed according to the manufacture’s procedure. Following the color development in all assays, the optical densities were measured on a microplate reader SpectraMax M2 (Molecular Devices, Sannyvale, CA, USA). As necessary, the supernatant samples were diluted and applied to the assay.

### Statistical analysis

Data are expressed as the mean+/− standard deviation. Statistical analysis was performed with an unpaired t-test when comparing two groups (Fig. 2B, Fig. 3A, and Fig. 5B). Multiple comparisons were analyzed by one-way ANOVA with the Tukey-Kramer HDS test (Fig. 5C). In Fig. 6, the EPS(+) and EPS(−) groups were compared by using one-way ANOVA and then only Fig. 6A and C were analyzed by the t-test for each of the days recorded. Statistical significance was considered as *P < 0.05 and **P < 0.01. Statistical processing was performed using JMP Software.

## Electronic supplementary material

Supplementary Information

Supplementary Video 1

Supplementary Video 2

Supplementary Video 3

Supplementary Video 4
