# Supplementary material for: Engineered Human Contractile Myofiber Sheets as a Platform for Studies of Skeletal Muscle Physiology
Source: Sci Rep. 2018 Sep 17;8:13932. doi: 10.1038/s41598-018-32163-1 (PMC6141563; doi:10.1038/s41598-018-32163-1)
Supplement: Supplementary file 1 — Supplementary Information [file 41598_2018_32163_MOESM1_ESM.docx]

**Supplementary Information**

**Engineered Human Contractile Myofiber Sheets as a Platform for Studies of Skeletal Muscle Physiology**

Hironobu Takahashi, Tatsuya Shimizu, and Teruo Okano

Institute of Advanced Biomedical Engineering and Science, Tokyo Women’s Medical University

**Supplementary Video 1.** EPS-induced twitch and tetanic contraction of a myofiber sheet at various frequencies (0.5, 1, 2, and 15 Hz). The video is shown at a field of view of 1066 μm x 800 μm.

**Supplementary Video 2.** EPS-induced twitch contraction of a myofiber sheet (stimulated at 1 Hz frequency) at 2 months after the induction of differentiation. The video is shown at a field of view of 1066 μm x 800 μm.

**Supplementary Video 3.** Representative change in EPS-induced muscle contraction of myofiber sheets after the addition of ryanodine (50 μM). Video is shown at a field of view of 1066 μm x 800 μm.

**Supplementary Video 4.** EPS-induced muscle contraction of myofiber sheets after 3 day-culture with and without continuous EPS (1 h EPS with 3 h rest). In the movies, all myofiber sheets were stimulated electrically at a frequency of 1 Hz to estimate the contractile abilities. (A) The myofiber sheets were cultured for 10 days without EPS under the normal culture conditions. (B) The myofiber sheets were first cultured for 7 days under normal conditions and then stimulated continuously at a frequency of 1 Hz for 3 days.
